# Population Pharmacokinetics of Temocillin Administered by Continuous Infusion in Patients with Septic Shock Associated with Intra-Abdominal Infection and Ascitic Fluid Effusion

**DOI:** 10.3390/antibiotics11070898

**Published:** 2022-07-05

**Authors:** Perrin Ngougni Pokem, Xavier Wittebole, Christine Collienne, Hector Rodriguez-Villalobos, Paul M. Tulkens, Laure Elens, Françoise Van Bambeke, Pierre-François Laterre

**Affiliations:** 1 Pharmacologie Cellulaire et Moléculaire, Louvain Drug Research Institute, Université Catholique de Louvain, 1200 Brussels, Belgium; perrin.ngougni@uclouvain.be (P.N.P.); paul.tulkens@uclouvain.be (P.M.T.); 2 Integrated PharmacoMetrics, PharmacoGenomics and PharmacoKinetics, Louvain Drug Research Institute, Université Catholique de Louvain, 1200 Brussels, Belgium; laure.elens@uclouvain.be; 3 Department of Critical Care Medicine, Cliniques Universitaires Saint-Luc, Université Catholique de Louvain, 1200 Brussels, Belgium; xavier.wittebole@saintluc.uclouvain.be (X.W.); christine.collienne@saintluc.uclouvain.be (C.C.); pierre-francois.laterre@saintluc.uclouvain.be (P.-F.L.); 4 Clinical Microbiology Department, Cliniques Universitaires Saint-Luc, 1200 Brussels, Belgium; hector.rodriguez@saintluc.uclouvain.be

**Keywords:** temocillin, intra-abdominal infection, ascitic fluid, population pharmacokinetics, Monte Carlo simulations

## Abstract

Temocillin is active against Gram-negative bacteria, including many extended-spectrum β-lactamase (ESBL)-producing Enterobacterales. We studied its pharmacokinetics in plasma and ascitic fluid after intravenous administration of a loading dose of 2 g over 30 min, followed by continuous infusion of 6 g/24 h, to 19 critically-ill patients with septic shock associated with complicated intra-abdominal infection. We established a pharmacokinetic model describing unbound temocillin concentrations in plasma and ascitic fluid and performed Monte-Carlo simulations to evaluate the probability of target attainment (PTA) of unbound concentrations (100% *f*T > MIC, i.e., unbound concentrations remaining above the MIC during 100% of the time) for the applied and hypothetical dosing regimens. The temocillin AUC in ascitic fluid was 46% of the plasma AUC. Plasma unbound concentrations were best described by a two-compartment model, and an additional compartment was added to describe unbound concentration in ascitic fluid, with renal clearance as a covariate. Dosing simulations showed that 90% PTA was achieved in the plasma with the current dosing regimen for MIC ≤ 16 mg/L (EUCAST susceptibility breakpoint) but not in the ascitic fluid if renal clearance was ≥40 mL/min. Hypothetical dosing with a higher (a) loading dose or (b) infused dose allowed to reach target concentrations in ascitic fluid (a) more rapidly or (b) sustainably, but these simulations need to be evaluated in the clinics for safety and efficacy.

## 1. Introduction

Intra-abdominal infections (IAI) in critically-ill patients are associated with high morbidity and mortality, making their treatment highly challenging [1]. Changes in the pathophysiology of patients during sepsis or septic shock lead to altered pharmacokinetics (PK) of antibiotics, further influencing the outcome of the treatment [2,3,4]. Therapeutic guidelines recommend a timely control of the source of the infection combined with rapid initiation of the right antibiotic [1,5,6]. An early intravenous empiric antibiotic therapy with a broad-spectrum antibiotic showing adequate penetration in the suspected site of infection largely contributes to a favorable outcome [1,7]. Nevertheless, the empirical use of a narrower spectrum antibiotic that covers the likely causative organisms, or, alternatively, a de-escalation of therapy from a broad to a narrow spectrum drug based on the results from the microbiological susceptibility testing, could be a desirable option for ecological reasons [8,9,10]. 

Advanced generation β-lactams are often first-line therapies for critically-ill patients based on their broad-spectrum, low toxicity, and high activity on Gram-negative bacteria [11], which represent the most common microorganisms isolated in IAI [12]. However, the growing incidence of resistant bacteria, notably extended-spectrum β-lactamase (ESBL)-producing Enterobacterales, sharply narrows treatment options [12,13].

Temocillin (6-methoxy-ticarcillin) is a β-lactam antibiotic active, among others, against Enterobacterales [14,15]. The interest in this molecule has been revived thanks to its stability to many extended-spectrum β-lactamases (ESBL), with minimum inhibitory concentrations (MICs) ranging from 2 to 32 mg/L [16,17,18,19]. In addition, it has no or limited impact on the human intestinal flora [20,21]. For these reasons, temocillin is considered a sparing drug for carbapenems [22,23]. It is currently licensed for use in septicemia, urinary tract, wound, and lower respiratory tract infections where susceptible Gram-negative bacilli are suspected or confirmed [24].

For β-lactam antibiotics, the PK/PD parameter driving efficacy consists of the time interval during which the unbound concentrations remain above the MIC against the target microorganisms (*ƒ*T > MIC) [25], but the value of this parameter (40% or 100% of the dosing interval, above 1 to 4 × the MIC) is still hotly debated [26]. For highly protein-bound drugs, including antibiotics, it is commonly admitted that the unbound drug is responsible for the activity [27,28,29]. In this context, it is important to note that temocillin shows a saturable and highly variable [30,31] plasma protein binding, ranging from around 85% in adult healthy volunteers [15,32] to a mean value of 59% (range: 19 to 85%) in critically-ill patients [33]. A previous PK study in critically-ill patients showed that a daily dose of 6 g given as continuous infusion allows to sustainably maintain unbound serum concentrations above 16 mg/L in the vast majority of the patients [34]. Yet, in critically-ill patients with intra-abdominal infection, supra MIC unbound concentrations at the site of infection are warranted [2,34]. More specifically, a consensus conference on the management of IAI recommends the use of a loading dose when indicated, especially in critically-ill patients, followed by extended or prolonged infusion for β-lactam antibiotics; it also advises selecting drugs with peritoneal distribution [1].

However, there are so far no data regarding the penetration of temocillin in the ascitic fluid. In other fluids, temocillin penetration usually reaches values ranging from 8–15% (in the cerebrospinal fluid [35]) to 50–70% (in peritoneal fluid, blister fluid, peripheral lymph, epithelial lining fluid [36,37,38,39]) or even 8–10 times higher than in serum (in the bile [40,41]). However, these studies did not differentiate between total and unbound concentrations and did not estimate the probability of reaching pharmacodynamic targets in these fluids. 

In this context, the present study was designed to model, using population PK approaches, the unbound temocillin concentrations in plasma and ascitic fluid of critically-ill patients during septic shock associated with complicated IAI, and to determine the penetration of temocillin in ascitic fluid, after intravenous administration of a loading dose of 2 g over 30 min, followed by continuous infusion of 6 g/24 h. This scheme of administration is recommended for severe infections in the Summary of Product Characteristics [42] and has been previously used to treat critically-ill patients in our institution [33]. The probability of target attainment (PTA, with a target set at 100% *f*T > MIC) was then estimated for MICs of 8 or 16 mg/L (current EUCAST limit of susceptibility [43]) and relevant patients’ clinical profiles, through Monte-Carlo simulations and using our validated population PK model.

## 2. Results

### 2.1. Study Population, Treatment Parameters, and Outcomes

Demographic and biological data are presented in Table 1. Nineteen patients in septic shock associated with IAI and ascitic fluid effusion (median and range for age: 56 years (21–74)) were enrolled in the study and contributed a total of 114 blood and ascitic fluid samples. Urinary creatinine clearance, plasma total protein, and albumin levels were low compared to normal values (median and range: 39.9 mL/min (20.5–149.3); 47.4 g/L (29.6–58.7); 22.3 g/L (13.7–30.8)). Significant amounts of proteins and albumin were measured in ascitic fluid (median and range: 11.6 mg/L (6.2–36.5); 5.3 mg/L (2.1–12.4)), but there was no correlation between protein or albumin levels in the ascitic fluid and in the plasma (Figure S1). SOFA and APACHE II scores were 9 (4–14) and 18 (13–32), respectively. All patients were treated for IAI with positive blood culture. All patients with spontaneous peritonitis were cirrhotic (Child-Pugh score: 10 (7–14); MELD score: 26 (13–38)). 

### 2.2. Microbiological Data

A total of 39 bacteria were isolated, among which *Escherichia coli* (n = 20) and *Klebsiella* spp. (n = 10) were the most frequent (Table 2). Temocillin MICs varied from ≤2 to 32 mg/L, with 97.5% being ≤16 mg/L. ESBLs and cephalosporinases were detected in 26.31% and 15.78% of the isolates, respectively.

### 2.3. Pharmacokinetic Analysis

The individual concentrations time-profiles of total and unbound temocillin in plasma and ascitic fluid are shown in Figure S2. Although the drug was administered by continuous infusion, individual profiles showed variations over time, even at a steady state. Thirty minutes after the loading dose, total and unbound concentrations reached 131.2 mg/L (5.3–160.2) and 85.9 mg/L (35.9–125.5) in plasma and 9.2 mg/L (3.4–35.2) and 3.0 mg/L (1.0–15.7) in ascitic fluid, respectively. The peak concentration in the ascitic fluid was reached between 12 and 96 h after the loading dose. The unbound fraction of temocillin in plasma and ascitic fluid were 56.4% (24.5–78.3%) and 57.4% (19.1–93.4), respectively. Penetration in ascitic fluid reached 46.0% (30.0–61.6%), corresponding to a proportion of active temocillin in the ascitic fluid of 23.0% (14.4–39.0%). There was no correlation between the penetration of temocillin into ascitic fluid and the concentration of total proteins and albumin in plasma and ascitic fluid, neither with a CRP or SOFA score, while a positive correlation was observed with the APACHE II score (Figure S3). A significant correlation was also evidenced between the area under the curve (AUC) of temocillin in plasma and in ascitic fluid considering both the total (r = 0.75; *p* = 0.0002) and the unbound (r = 0.80, *p* < 0.0001) concentrations as well as between the unbound plasma AUC of and the total AUC in ascitic fluid (r = 0.71, *p* = 0.0006) (Figure S4).

### 2.4. Population Pharmacokinetic Modelling

**Figure 1:**
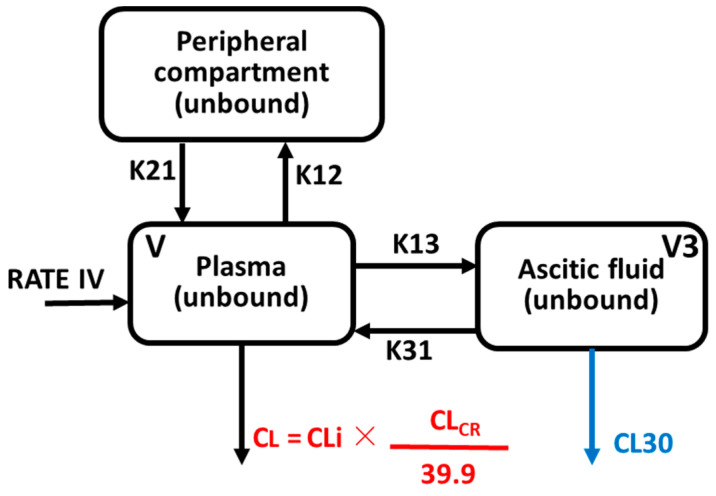
Graphical representation of PK final model: V (L), the volume of the central compartment; CLi (L/h), population parameter estimate of temocillin clearance from central compartment; CL (L/h), typical estimate of clearance from central compartment; K12 (h^−1^), first-order rate constant for distribution from central to peripheral compartment 2; K21 (h^−1^), first-order rate constant for distribution from peripheral compartment 2 to central compartment; K13 (h^−1^), first-order rate constant for distribution from central to peripheral compartment 3 (ascitic fluid compartment); K31 (h^−1^), first-order rate constant for distribution from peripheral compartment 3 to central compartment; V3 (L), volume of the compartment 3; CL30 (L/h), clearance from compartment 3 defined as the product between of first-order elimination rate constant from compartment 3 (K30 (h^−1^)) and volume of the compartment 3 (V3 (L)).

PK modelling was performed using the data from the 114 plasma and ascitic fluid unbound concentrations. We limited our modeling to the study of the unbound concentrations, which are considered responsible for antimicrobial activity. The structure of the final covariate model is presented in Figure 1 whereas the model template is detailed in Table S1. 

The plasma unbound concentration of temocillin was best described by a two-compartment model, as previously published [32], and an additional compartment was added to describe unbound concentration in ascitic fluid (-2LL = 1497, AIC = 1514). Ascitic fluid was eliminated via a drain, thus, non-renal elimination from this additional compartment (CL30) was associated with a significant reduction of -2LL and AIC (Δ-2LL = 10, AIC = 1506). Temocillin plasma clearance (normalized to its median value for the study population as CL = CLi × (CL_CRurinary_/39.9) where CLi is the population estimate of temocillin clearance from the central compartment, and CL is the individual estimate of temocillin clearance from the central compartment) for a given patient and was linearly related to urinary creatinine clearance (CL_CRurinary_) (Figure S5). CL_CRurinary_ was therefore included as a covariate to improve model fit (better diagnostic plots, minimization of bias and imprecision, but a non-significant reduction of -2LL and AIC [-2LL = 2, AIC = 1504]). Model diagnostics and selection criteria are presented in Table 3. 

No linear relationship was observed between weight, age, plasma proteins, or albumin and CL30, neither between CL30 and Vd, Cli, or CLs (R^2^ values between 0 and 0.24). 

**Figure 2:**
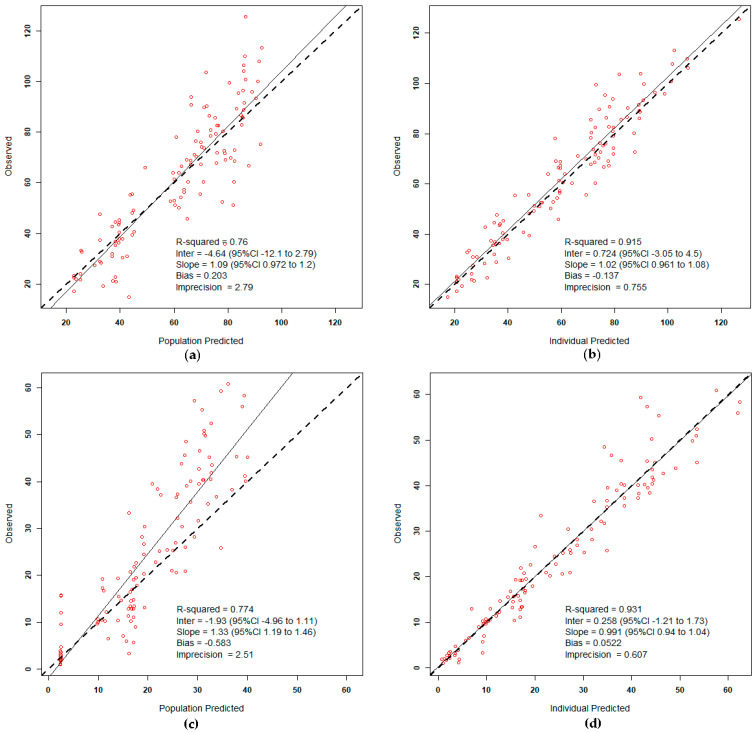
Scatter plots showing observed-versus-predicted values for the population PK model after the Bayesian step. The different panels show the population predicted concentrations in plasma (**a**) and ascitic fluid (**c**), and the individual posterior predicted concentrations in plasma (**b**) and ascitic fluid (**d**).

For the error model, each observation was weighted by 1/Error^2^ with Error = (SD + L^2^)^0.5^, where L (lambda factor) is the process noise associated with the observations. The final Lambda (L) error factor was set at 2.26 for the residual unexplained source of variability. Residual error or uncertainty associated with the assay was best described by first-order polynomial functions: SD = 0.1 + 0.1Y, where SD is the standard deviation of measured temocillin concentrations (Y), for both plasma and ascitic fluid. The population PK parameter estimates for the final model are presented in Table 4. The observed versus predicted diagnostic plots for the final models indicate adequate fitting of the model to the data as shown in Figure 2. 

**Figure 3:**
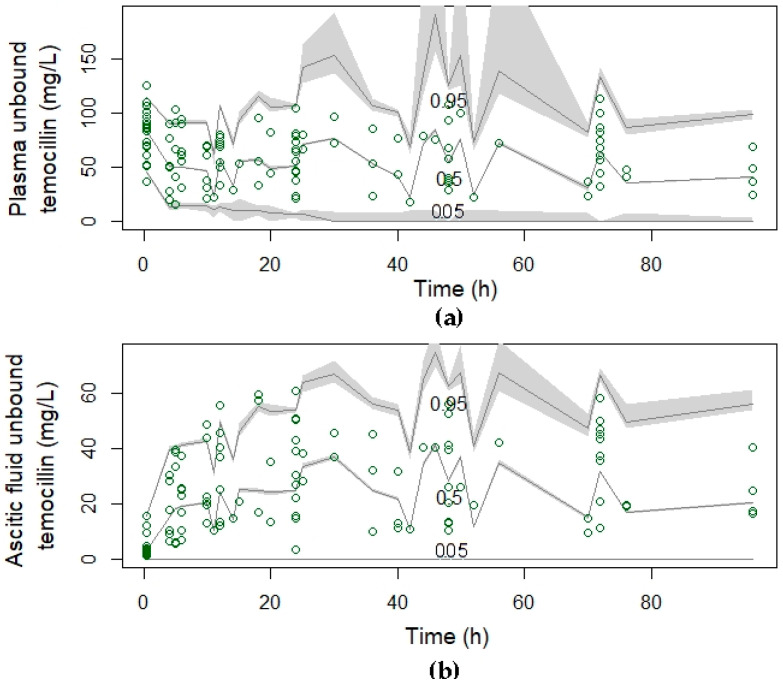
Visual predictive checks of the final covariate model for unbound temocillin in plasma (**a**); central compartment or ascitic fluid (**b**). The lines represent the percentiles of 1000 simulated temocillin concentration-time profiles superimposed with observed temocillin concentrations (green circles) after a loading dose of 2 g of temocillin over a 30 min infusion, followed by a continuous infusion of 6 g/24 h. The grey shading around the lines represents the 95% CI around each percentile. The distribution of the simulated unbound temocillin concentration profiles is similar to that of the observed unbound temocillin concentrations, with 100% of the observed concentrations found between the 5th and 95th simulated percentiles, suggesting that the model describes the data adequately.

Visual inspection of the residual plots is shown in Figures S6 and S7 for unbound temocillin in plasma and ascitic fluid, respectively. The error of the weighted residuals appeared to be evenly distributed around the population’s predicted concentrations, and around time, centered at zero and along with a normal frequency distribution (D’Agostino test, *p* = 0.714; Shapiro–Wilk test, *p* = 0.514; Kolmogorov-Smirnov test, *p* = 0.096 for plasma temocillin and D’Agostino test, *p* = 0.371; Shapiro–Wilk test, *p* = 0.041; Kolmogorov-Smirnov test, *p* = 0.797 for ascitic fluid temocillin). The visual predictive check plots, which highlight the performance, robustness, and acceptable agreement between the predicted and observed unbound concentrations of temocillin in plasma and ascitic fluid over the dosing interval, are presented in Figure 3 and indicate good concordance between simulated and observed unbound concentrations in both plasma and ascitic fluid (<5% outliers).

### 2.5. Probability of Target Attainment (PTA)

**Figure 4:**
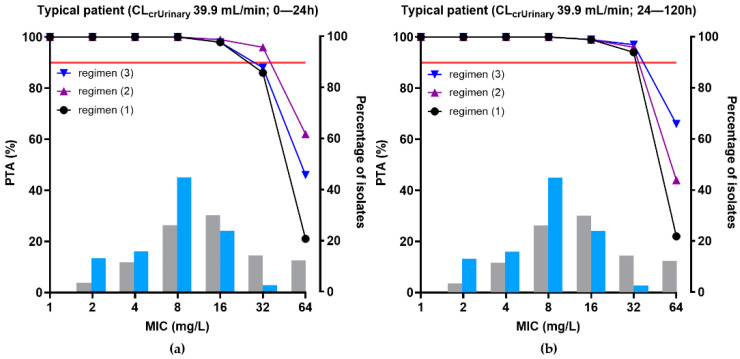
PTA of temocillin in plasma for typical septic patients (Median CL_CRurinary_ = 39.9 mL/min) with IAI and ascitic fluid effusion, for different MIC values. The following dosing regimens were simulated: (1) 2 g loading dose over 30 min infusion followed by a continuous infusion of 6 g/24 h; (2) 4 g loading dose over 30 min followed by a continuous infusion of 6 g/24 h; (3) 2 g loading dose over 30 min followed by a continuous infusion of 8 g/24 h. The PK/PD target is 100% fT> MIC; the PK/PD breakpoint corresponds to a PTA ≥ 90%. (**a**) PK profiles over the first 24 h; (**b**) PK profiles between 24 and 96 h. Blue and grey histograms: MIC distribution of the isolates of this study and of EUCAST for *E. coli* and *K. pneumoniae*, respectively.

**Figure 5:**
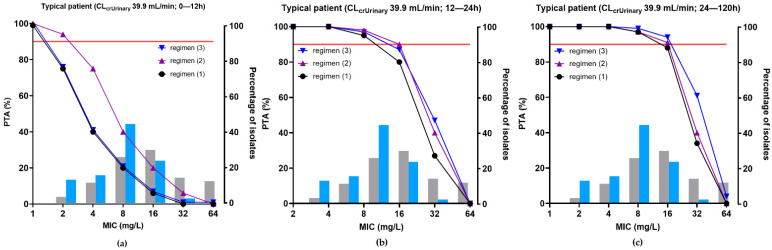
PTA of temocillin in ascitic fluid for typical septic patients (Median CL_CRurinary_ = 39.9 mL/min) with IAI and ascitic fluid effusion, for different MIC values. The following dosing regimens were simulated: (1) 2 g loading dose over 30 min infusion followed by continuous infusion of 6 g/24 h; (2) 4 g loading dose over 30 min followed by continuous infusion of 6 g/24 h; (3) 2 g loading dose over 30 min followed by continuous infusion of 8 g/24 h. The PK/PD target is 100% *f*T > MIC; the PK/PD breakpoint corresponds to a PTA ≥ 90%. (**a**) PK profiles over the first 12 h; (**b**) PK profiles between 12 and 24 h; (**c**) PK profiles between 24 and 96 h. Blue and grey histograms: MIC distribution of the isolates of this study and of EUCAST for *E. coli* and *K. pneumonia*e, respectively.

The PTA for achieving 100% *f*T > target MIC of unbound temocillin in plasma and in ascitic fluid for different simulated dosing regimens of temocillin in representative patients with septic shock and IAI (with a CL_CRUrinary_ of 39.9 mL/min) are illustrated in Figure 4 and Figure 5, respectively. In addition to the therapeutic scheme used to treat the patients (regimen (1), a 2 g loading dose over a 30 min infusion followed by a continuous infusion of 6 g/24 h, we simulated two hypothetical schemes. In regimen (2) (4 g loading dose over 30 min followed by a continuous infusion of 6 g/24 h), the loading dose was increased in order to evaluate whether it allows reaching the target in ascitic fluid earlier. In regimen (3) (2 g loading dose over 30 min followed by a continuous infusion of 8 g/24 h), the dose used during the continuous infusion was increased to explore whether it allows reaching the pharmacodynamic target for higher MICs. In plasma, the three therapeutic schemes allowed to reach a PTA > 90% for isolates with MICs ≤ 16 mg/L both at early time points (0–24 h) and at a steady-state (Figure 4a,b; Table 5). 

In contrast, in ascitic fluid, a PTA of 90% was achieved only for MICs < 2 mg/L during the first twelve hours when the loading dose was 2 g (regimens (1) and (3)) and for MICs < 4 mg/L when the loading dose was increased to 4 g (regimen (2)) (Figure 5a). During the next twelve hours, a PTA of 90% was achieved for MICs ≤ 8 mg/L for regimens (1) and (3), and ≤ 16 mg/L for regimen (2) (Figure 5b). At steady-state and for MICs of 16 mg/L, PTAs reached 97, 97, and 99% for regimens (1), (2), and (3), respectively (Figure 5c; Table 5 and Table 6). Higher CL_CRurinary_ was associated with a reduced PTA in ascitic fluid, but with no major impact in plasma (Table 5 and Table 6; Figure S8). 

## 3. Discussion

This study is the first to describe the pharmacokinetics of temocillin administered by continuous infusion in plasma and ascitic fluid from patients with septic shock associated with IAI and ascitic fluid effusion. Our main conclusion is that an infusion of 6 g/24 h after a loading dose of 2 g allows for maintaining the unbound temocillin concentration in the plasma above a MIC of 16 mg/L 100% of the time whereas, in the ascitic fluid, this regimen seems to be adequate only for patients with altered renal function. In addition, a series of observations with clinical implications have been made. 

First, the direct examination of individual pharmacokinetic profiles reveals high variability in the total and unbound concentrations reached both in the plasma and in the ascitic fluid, not only between patients but also over time in a given individual patient, in spite of the fact they all received the same dose by continuous infusion. This variability was also found in the population PK model constructed from these data. The PK profile of unbound temocillin was best described by a two-compartment model with an additional distribution/elimination compartment corresponding to ascitic fluid. The inter-individual variation was greater than 50% for inter-compartmental clearances as well as for the volume of distribution of the ascitic fluid compartment. This variation was expected and is in the line of previous observations with continuous infusion of temocillin or other antibiotics in critically-ill patients. It might be explained by the variability and instability of the patient’s pathophysiological and biochemical characteristics over time [44,45]. For example, in patients with sepsis or septic shock, blood flow parameters are altered, which can lead to altered tissue distribution or impaired kidney function [46]. Importantly, fluctuations in albumin/protein levels may also influence the proportion of the drug bound to proteins, as well-documented for highly protein-bound β-lactams [31,47,48]. 

Second, temocillin adequately penetrates the ascitic fluid, with an ascitic fluid/plasma AUCs ratio of 46%; this is slightly lower than that previously reported for ceftazidime (67%) or ceftriaxone (63%) in patients with cirrhosis or peritoneal carcinoma but normal renal function [49] or for temocillin in the peritoneal fluid (60%) of patients receiving elective gastrointestinal surgery [38]. In these studies, the drugs were given by discontinuous infusion. Whether this contributes to explaining why a longer time to reach the maximal concentration (12 to 72 h vs. 1 to 4 h) in ascitic fluid as observed in our study remains to be established. Another critical property governing temocillin diffusion among compartments is its protein binding. It is worth observing that in the majority of the patients (15/19), the total concentration of temocillin in ascitic fluid and the unbound concentration in plasma are very close to one another once the steady-state has been achieved, suggesting a high degree of diffusibility from the blood to the ascitic fluid. Intriguingly, however, the unbound fraction of temocillin in plasma and ascitic fluid is similar (56–57%) in spite of the lower protein and albumin concentrations in the ascitic fluid (23% of the plasma concentrations). Based on the saturable character of temocillin protein binding [31], higher unbound fractions would have been expected in ascitic fluid, as described for ceftriaxone [50]. A possible explanation for this divergence could reside in differences in the binding capacity or affinity in the ascitic fluid vs. the plasma, possibly due to differences in the protein composition [51] or in the physicochemical properties (including a slightly more acidic pH in the infected ascitic fluid [52]) of these liquids. 

When comparing this penetration in ascitic fluid with that reported in a series of other body fluids (peritoneal fluid, blister fluid, peripheral lymph, epithelial lining fluid [36,37,38,39]), values of the same order of magnitude or slightly higher (50–70%) are obtained, confirming temocillin capacity to diffuse in these liquids. Regarding tissue penetration, limited data reports concentrations in the prostate reaching 26–35% of total plasma concentrations [53], but a concentration higher in a pancreatic biopsy than in the plasma [54]. Unbound concentrations were also higher in the subcutis and muscle of healthy volunteers than in the plasma [32]. Altogether, these data suggest that temocillin can also get access to tissues and organs, which could be useful for tissular infections. Here, bacteria were isolated from ascitic fluid, hemocultures, and less frequently, from abdominal pus or urine, so that plasma and ascitic fluid concentrations could be used in Monte-Carlo simulations to estimate PTA. 

Monte Carlo simulations showed that exposure to temocillin in the ascitic fluid of a representative patient (CL_CRurinary_ 39.9 mL/h) was excellent when given as a continuous infusion (CI) of 6 g/24 h after a loading dose of 2 g; regimen (1)), leading to a PTA ≥ 88% considering a pharmacodynamic target of unbound concentration above a MIC ≤ 16 mg/L 100% of the time. This target was, however, not reached over the first 24 h, or during the first 12 h even if doubling the loading dose (regimen (2)). This could be problematic, as an early effective treatment increases the chance of clinical success [1,6]. A renal function higher than the median value of our population was identified as a risk factor for non-attainment of the pharmacodynamic target in ascitic fluid, in accordance with the fact that increased renal clearance is recognized as an important risk factor for low concentrations of β-lactam antibiotics in plasma and tissues, including when given by continuous infusion [55,56,57]. This negative effect can be partially corrected by increasing the infused dose (regimen (3); 8 g/24 h) but the benefit remained limited to patients with patients < 90 mL/min. Even higher doses would be required on a pharmacodynamic basis for patients with higher CL_CRurinary_, but would also generate sustained total plasma concentrations higher than the current peak level measured after discontinuous infusion, requiring prior in-depth safety assessment. Of note, a large proportion (72%) of the isolates had MICs ≤ 8 mg/L, allowing them to reach the pharmacodynamic target with the regimen (1) for CL_CRurinary_ < 90 mL/min. In this context, it is interesting to note that microbial eradication was obtained in 84% of the patients. 

In contrast, in the plasma, unbound concentrations, which are higher than those measured in ascitic fluid, allow reaching the pharmacodynamic target for MIC ≤ 16 mg/L with the conventional dosing regimen (1), whatever the renal function of the patient, as observed in a previous cohort of critically-ill patients [33]. Importantly, lower pharmacodynamic targets (4 or 8 mg/L depending on the creatinine clearance) were reached using the same dosing regimen (1) in critically-ill patients with pneumonia in a study by Layios et al. [39]. The plasma unbound temocillin concentrations reported at a steady-state by these authors were approximately five-fold lower than those observed here (mean values with SD: 13.7 ± 11.8 mg/L [39] vs. 61.8 ± 25.7 mg/L). This discrepancy can probably be explained by higher creatinine clearance in their population (mean values with SD: 119.2 ± 33.1 mL/min [39] vs. 58.1 ± 37.4 mL/min). Moreover, the study of Layios et al. does not report the plasma protein/albumin levels in their patients, which are critical determinants of the unbound fraction [31].

We acknowledge some limitations of this work. The number of included patients remained limited but was still sufficient to establish a valid population PK model and run robust Monte-Carlo simulations. However, the study protocol did not anticipate dose adjustments based on PK data and we were not able to test our recommendations. Moreover, the study was not powered enough to evaluate the treatment’s clinical efficacy and to correlate it with PK/PD markers. Nevertheless, our data might help in guiding the design of further studies by taking these limitations into account.

At this stage, we can however already conclude that the clearance and the PTA of unbound temocillin in critically-ill patients with intra-abdominal infection are mainly dependent on CL_CRUrinary_. The currently used regimen (2 g loading dose, followed by continuous infusion of 6 g/24 h) allows to achieve adequate PTA for isolates with MICs below the EUCAST resistance breakpoint of 16 mg/L in plasma, and in the ascitic fluid of patients with CL_CRUrinary_ < 40 mL/min. Dose adjustments are proposed but would need to be clinically evaluated, especially regarding the safety of this dose escalation. Indeed, only limited data in healthy volunteers are available so far with increased dosing regimens. They failed to detect safety issues after 8 days of treatment with 4 g twice daily [58]. This dose reproduces the increased loading dose from the regimen (2) simulated here but not yet the sustained elevated levels generated in the simulated regimen (3), thus calling for caution before applying these schemes. Nevertheless, the fact that temocillin penetration in ascitic fluid is high is reinsuring regarding its potential activity in peripheral body compartments, at least for microorganisms with sufficiently low MICs. 

## 4. Materials and Methods

### 4.1. Study Design, Patients, and Data Collection

This prospective, monocentric, open-label, and non-randomized pharmacokinetic study enrolled adult patients with septic shock associated with intra-abdominal infection (IAI) hospitalized in the intensive care unit of the *Cliniques universitaires St-Luc* (Brussels, Belgium). The inclusion criteria were patients with septic shock, >18 years old, diagnosed with an IAI caused by a pathogen expected to be susceptible to temocillin. The exclusion criteria were patients allergic to any penicillin, including temocillin, pregnant or lactating women; or patients having participated in another experimental study with the same drug during the 4 preceding weeks.

The following parameters were recorded in all patients: demographic data (age, gender, weight, body mass index), treatment duration, isolated pathogens, severity scores (acute sepsis-related organ failure assessment (SOFA) [59], and Acute Physiology, Age, Chronic Health Evaluation (APACHE II) [60]), medical history, biological and physiological parameters (urinary creatinine clearance (CL_Crurinary_, C-reactive protein, total protein and albumin levels in plasma and ascitic fluid, hepatic enzymes serum levels (GGT, ALAT, and ASAT). A Child-Pugh score [61] and Model for End-Stage Liver Disease score (MELD) [62] were calculated for patients with spontaneous peritonitis.

### 4.2. Antibiotic Treatment and Sample Collection

All patients were treated with temocillin according to the following scheme: a loading dose (2 g) was administered over 30 min in 50 mL of water for injection, followed by a continuous infusion (6 g/24 h in 48 mL of water for injection infused at a rate of 2 mL/h). Temocillin was given as monotherapy for documented infections caused by susceptible pathogens. Additional antibiotics were given for Gram-positive bacteria as needed. Blood and fresh ascitic fluid samples were drawn between 0.5 to 96 h after the start of the treatment (Figure S9). All blood samples (5 mL) were drawn with an arterial catheter, collected in EDTA tubes, and centrifuged at 2000× *g* for 15 min at 4 °C. All fresh ascitic fluid samples (10 mL) were collected via the drainage system in a tube (without anticoagulant, clot activator, or gel), simultaneously with each blood sample when possible. All plasma and ascitic fluid samples were stored at −80 °C until analysis. 

### 4.3. Analytical Method

#### 4.3.1. Chemicals and Reagents 

Temocillin was obtained from EUMEDICA S.A., Manage, Belgium, as the branded product (NEGABAN) approved for parenteral human use in Belgium, the UK, and France. Ticarcillin disodium (internal standard, IS) was acquired from Sigma-Aldrich Corp., St. Louis, MO, USA; HPLC-grade methanol and acetonitrile, from J.T. Baker, Deventer, The Netherlands; formic acid and ammonium acetate, from Merck KGaA, Darmstadt, Germany. Ultrapure water was from a MEDICA-R 7/15 water purification system (Veolia Water Systems, High Wycombe, UK) or a Milli-Q Academic apparatus (Merck-Millipore, Darmstadt, Germany). 

#### 4.3.2. Temocillin Assay

Total and unbound (free) temocillin plasma and ascitic fluid concentrations were measured by an HPLC-MS/MS method, previously validated for assay in serum, plasma, and ascitic fluid [32,63,64] using ticarcillin as an internal standard. Total concentrations were measured after methanol precipitation, and unbound concentrations, on ultrafiltrates (exclusion cut-off: 30 kDa). Calibration curves showed that the assay was linear in both plasma and ascitic fluid over a range of concentrations covering those measured in clinical samples (1–500 mg/L and 0.75–300 mg/L, respectively, for the total and unbound temocillin concentrations in plasma, and 1–150 mg/L and 1–100 mg/L, respectively, for the total and unbound temocillin concentrations in ascitic fluid). 

### 4.4. Microorganisms and Minimum Inhibitory Concentrations (MIC) Determinations

Microorganisms were identified and MIC determined using automated routine systems available at the clinical microbiology laboratory (MALDI-TOF MS, Phoenix^®,^ and E-test^®^) of the *Cliniques universitaires Saint-Luc.*

### 4.5. Pharmacokinetic Analysis

Plasma and ascitic fluid total and unbound temocillin concentrations were plotted against time and the area under the concentration-time curve (AUC) was determined [65]. Unbound fraction (UF) in plasma and ascitic fluid, percentage of penetration (PE) and active proportion (PR) of temocillin in the ascitic fluid were calculated as UF (%) = 100 × (C_unbound_/C_total_); PE (%) = 100 × (AUC_total in ascitic fluid/_AUC_total in plasma_), and PR (%) = 100 × (AUC_unbound in ascitic fluid_/AUC_total in plasma_), respectively.

### 4.6. Population Pharmacokinetic Modeling

#### 4.6.1. Model Building

Modeling was performed only for unbound concentrations because these are those considered microbiologically active. We used experimentally measured unbound concentrations and did not calculate them based on total concentrations, which would be rather complex because total and unbound concentrations are not linearly related due to the saturable, concentration-dependent character of temocillin protein binding. Unbound temocillin in plasma and ascitic fluid were co-modeled using the non-parametric adaptive grid (NPAG) algorithm within the Pmetrics^®^, version 1.5.1 package for R (Los Angeles, CA, USA) [66]. One-, and two-compartment models with first-order elimination from the central compartment and inter-compartmental distribution were first tested to fit plasma unbound temocillin concentrations, and an additional compartment was used to fit the ascitic fluid unbound temocillin concentrations, with an additional non-renal elimination from this compartment corresponding to the drain. This non-renal elimination was defined by its elimination constant, calculated as the ratio between the clearance and the volume of distribution for this compartment. For the error model, each observation was weighted by 1/Error^2^ with Error = (SD + L^2^)^0.5^ and Error = SD × G, where L (lambda factor) and G (gamma factor) are process noises associated with the observations. In addition, the error associated with the analytical assay was modelled as a second-degree polynomial function (SD = C_0_ + C_1_Y + C_2_Y^2^ + C_3_Y^3^), where SD is the standard deviation of measured temocillin concentrations (Y), and C_0_, C_1_, C_2_, and C_3_ are coefficients calculated from assay validation data supplied by the analytical method of the dosage of temocillin in plasma and ascitic fluid, or independently estimated by Pmetrics^®^.

#### 4.6.2. Covariate Exploration and Model

Demographic and biologically plausible covariates were screened in Pmetrics^®^ using univariate associations between the tested covariate and individual median Bayesian estimates of PK parameters such as volume of distribution of the central compartment (V_c_) and clearance (CL). Covariates selected for screening included age, weight, height, body mass index, C-reactive protein, urinary creatinine clearance, plasma, and ascitic fluid albumin level, SOFA, and APACHE II scores. After the selection of significant covariates in univariate analysis, a covariate model was built using stepwise forward inclusion followed by backward elimination if necessary. At each step of inclusion, the model with the greatest reduction in the -2LL and/or improved goodness-of-fit plots was retained.

#### 4.6.3. Model Diagnostics and Selection

Model diagnostics included goodness-of-fit of the observed versus predicted plots, minimization of bias and imprecision, shrinkage, the precision of PK parameter estimates, Akaike information criterion (AIC), and satisfactory visual predictive checks (VPC), and consideration and the −2log-likelihood ratio test (-2LL). The -2LL ratio test was chosen for the selection between two hierarchical models. The difference in -2LL of 2 hierarchical models follows approximately a χ2 distribution so that a decrease of 3.84 in the -2LL was considered statistically significant (*p* < 0.05).

#### 4.6.4. Model Validation

The predictive performance and robustness of all PK parameter estimates in the final model were assessed through Monte-Carlo simulation. From a joined parameter probability distribution using NPAG, the simulator in Pmetrics^®^ draws random samples repeatedly. A thousand simulated profiles for each subject using their own set of covariates, dose, and sampling schedule were created from the final population model parameters. The predictive performance of the model was determined using VPC comparing simulation results and observations.

#### 4.6.5. Dosing Simulations and Probability of Target Attainment 

Monte-Carlo dosing simulations (n = 1000) were performed for temocillin regimens of (1) 2 g loading dose infused 30 min followed by continuous infusion of 6 g/24 h; (2) 4 g loading dose over 30 min followed by continuous infusion of 6 g/24 h; (3) 2 g loading dose over 30 min followed by continuous infusion of 8 g/24 h. Dosing simulations were performed from 0 to 12 h, 12 to 24 h, and 24 to 96 h. The pharmacodynamic target recommended by EUCAST for temocillin is to maintain unbound concentrations above the MIC 35–41% of the time (%*f*T > MIC of 35–41%), with a resistance breakpoint set at MIC >16 mg/L [44]. Considering that patients included in this study were critically-ill and that temocillin was administered by continuous infusion, we rather aimed at determining the dose allowing us to achieve 100% *f*T > target MIC of temocillin in plasma and ascitic fluid throughout the treatment. An a priori probability of target attainment (PTA) of ≥90% was considered optimal [67]. 

### 4.7. Statistical Analysis

Statistical analyses were performed using version 9.3.1. of GraphPad software (GraphPad Prism Software, San Diego, CA, USA). The used parametric or non-parametric tests are indicated in the text, based on the preliminary determination of the normality of the distribution by the Shapiro–Wilk test. In all cases, the results were considered statistically significant when the *p*-value is less than 0.05. Data are expressed in median and [range] unless otherwise specified.

## Figures and Tables

**Table 1:** Demographics and characteristics of patients.

Parameter		Value (Median (Range)) ^a^
Patients enrolled, n		19
**Demographic data**		
Males, n (%)		6 (31.58%)
Age (years)		56 (21–74)
Weight (kg)		67 (45–95)
Body mass index (kg/m^2^)		23.87 (15.03–33.65)
**Biological and physiological parameters [local normal values]**		
C-reactive protein (mg/L) [<5 mg/L]		114.2 (20.00–364.6)
CL_CRurinary_ (mL/min) [>78 mL/min]		39.90 (20.55–149.3)
Plasma	Total protein (g/L) [64–83 g/L]	47.35 (29.59–58.74)
	Albumin (g/L) [35–52 g/L]	22.30 (13.70–30.80)
Ascitic fluid	Total protein (g/L)	11.64 (6.23–36.46)
	Albumin (g/L)	5.30 (2.12–12.45)
Gamma-glutamyl-transferase (IU/L) [<40 UI/L]		51.00 (13.0–205.0)
Alanine aminotransferase (IU/L) [7–35 UI/L]		34.00 (5.00–120.0)
Aspartate aminotransferase (IU/L) [9–36 UI/L]		49.00 (14.00–198.0)
Alkaline phosphatase (IU/L) (40–130)		119.0 (39.00–440.0)
Bilirubin—total (mg/dL) [<1.2]		3.3 (0.2–12.8)
Bilirubin—conjugated (mg/dL) [<0.3]		4.3 (0–11.9)
INR [0.80–1.20]		1.55 (1.09–4.7)
**Clinical scores at admission**		
SOFA score		9 (4–14)
APACHE II score		18 (13–32)
**Type of infection**		
Spontaneous bacterial peritonitis, n (%)		11 (58%)
Secondary peritonitis, n (%)		4 (21%)
Pancreatic infected necrosis, n (%)		3 (16%)
Liver abscess, n (%)		1 (5%)
**Temocillin treatment duration** (days)		5 (4–21)
**Outcome**		
Microbial eradication, n (%)		16 (84.21%)
Death, n (%)		7 (36.84%)

^a^ unless otherwise specified. Abbreviations: CL_CRurinary_, measured urinary creatinine clearance; SOFA, Sepsis-related Organ Failure Assessment; APACHE II: Acute Physiology, Age, Chronic Health Evaluation II.

**Table 2:** Microbiological data.

Type of Sample	Bacterial Species ^a^	Number of Isolates with a MIC (mg/L) ^b^					Detected β-Lactamase	
		≤2	3–4	6–8	12–16	>16	ESBL	Cephalos-Porinase
Ascitic fluid	*E. coli*		2	4	2		1	2
	*K. pneumoniae*	1		2	1		1	1
	*E. cloacae*	1						1
	*P. mirabilis*			1				
	*E. aerogenes*			1				
Abdominal pus/necrosis	*E. coli*	1	1	2	2		3	
	*K. pneumoniae*			1				
	*S. marcescens*				1			1
Hemoculture	*E. coli*		1	3			2	
	*K. pneumoniae*	2		1	1		2	1
	*K. oxytoca*		1					
	*P. mirabilis*		1					
	*S. marcescens*				1			
Urine	*E. coli*				1	1		
	*K. pneumoniae*			1				
	*K. oxytoca*			1			1	
Total, n (%)		5 (13.15)	6 (15.78)	17 (44.73)	9 (23.84)	1 (2.63)	10 (26.31)	6 (15.78)

^a^ MALDI-TOF MS; ^b^ as determined by Phoenix^®^ and E-test^®^; abbreviations: MIC, minimum inhibitory concentration; ESBL, extended-spectrum β-lactamase.

**Table 3:** Model diagnostics and selections.

Model	-2LL ^a^	AIC ^a^	Sample	R^2 b^	Slope ^b^ (95% CI)	Intercept ^b^ (95% CI)	Bias ^b^	Imprecision ^b^
Simple two-compartment	1860	1870	Plasma	0.812	0.891 (−0.99 to 1.13)	16.7 (−8.64 to −1.06)	0.54	1.46
			Ascitic fluid	0.87	0.68 (0.81 to 0.95)	5.64 (0.11 to 4.36)	0.40	1.45
Two-compartment + additional distribution compartment ^c^	1497	1514	Plasma	0.90	1.06 (−0.99 to 1.13)	−4.26 (−8.64 to −1.06)	0.21	1.26
			Ascitic fluid	0.84	0.88 (0.81 to 0.95)	2.24 (0.11 to 4.36)	0.20	1.21
Two-compartment + additional elimination compartment ^d^	1487	1506	Plasma	0.86	1.01 (0.0.93 to 1.08)	−0.14 (−5.46 to 1.90)	0.11	2.39
			Ascitic fluid	0.91	1.00 (0.94 to 1.06)	0.02 (−1.69 to 1.73)	0.09	1.07
**Two-compartment** **+ additional elimination compartment ^d^** **+ covariate ^e,f^**	**1485**	**1504**	**Plasma**	**0.92**	**1.02** **(0.96 to 1.08)**	**0.72** **(−3.05 to 4.50)**	**−0.13**	**0.75**
			**Ascitic fluid**	**0.93**	**0.99** **(0.94 to 1.04)**	**0.25** **(−1.21 to 1.73)**	**0.05**	**0.60**

^a^ -2LL, -2 log-likelihood; AIC, Akaike Information Criterion; ^b^ Result of the regression line fitted for the observed vs. predicted temocillin concentration after the Bayesian step. R^2^, R-square of linear regression; 95% CI, 95% confidence interval. ^c^ Additional compartment for drug distribution (ascitic fluid). ^d^ Additional compartment for drug distribution and non-renal elimination (ascitic fluid). ^e^ Allometric scale covariate model on CL, CL = Cli × (CL_Crurinary_/39.9). Cli (L/h), population parameter estimate of temocillin clearance from the central compartment; CL (L/h) typical estimate of clearance from the central compartment; V, the volume of distribution from the central compartment. ^f^ Bold: final model.

**Table 4:** Parameter estimates for unbound temocillin from the final covariate two-compartment Pop-PK model.

Parameter ^a,b^	Mean	SD	CV (%)	Median (95% CI)	Shrink (%)
V (L)	14.36	4.18	29.15	13.90 (11.76–15.98)	0.898
CLi (L/h)	2.45	0.91	37.33	2.56 (2.34–3.71)	0.235
K_12_ (h^−1^)	4.95	2.89	58.47	4.62 (2.93–6.53)	6.539
K_21_ (h^−1^)	5.38	3.62	67.20	5.85 (2.08–8.85)	6.677
K_13_ (h^−1^)	0.42	0.47	110.67	0.24 (0.16–0.41)	0.039
K_31_ (h^−1^)	0.33	0.46	137.94	0.15 (0.04–0.26)	0.010
V_3_ (L)	30.00	16.76	55.87	28.93 (15.83–42.77)	0.567
CL_30_ (L/h)	2.91	1.42	48.75	2.94 (2.11–3.60)	1.98

^a^ definition of parameters: V (L), volume of the central compartment; CLi (L/h), population parameter estimate of temocillin clearance from central compartment; K12 (h^−1^), first-order rate constant for distribution from central to peripheral compartment 2; K21 (h^−1^), first-order rate constant for distribution from peripheral compartment 2 to central compartment; K13 (h^−1^), first-order rate constant for distribution from central to peripheral compartment 3 (ascitic fluid compartment); K31 (h^−1^), first-order rate constant for distribution from peripheral compartment 3 to central compartment; V3 (L), volume of the compartment 3; CL30 (L/h), clearance from compartment 3 defined as the product between of first-order elimination rate constant from compartment 3 (K30 (h^−1^)) and volume of the compartment 3 (V3 (L)). ^b^ no statistical influence of GFR, body weight, age, or sex on these parameters (except sex on CL_30:_ mean value: 3.04 L/h [CI: 2.22–3.86] in females vs. 1.38 L/h [CI: 0.66–2.1]) in males (Mann-Whitney test: *p*: 0.022).

**Table 5:** Probability of target attainment (PTA) ^a^ (in percentages) in plasma between 0 to 96 h for the various temocillin dosing regimens according to the CL_CRurinary_ values for a target MIC of 8 and 16 mg/L.

Target MIC (mg/L)	CL_CRurinary_ (mL/min)	Temocillin Doses ^b^		
		Studied	Simulated	
		Regimen (1)	Regimen (2)	Regimen (3)
8 mg/L	20	100	100	100
	39.9	100	100	100
	60	100	100	100
	90	100	100	100
	120	100	100	100
	150	100	100	100
16 mg/L	20	98	99	98
	39.9	98	99	98
	60	98	99	98
	90	98	99	98
	120	98	99	98
	150	90	94	98

^a^ Dosing regimen achieving a priori target of PTA ≥ 90% are appearing on a green background. ^b^ Dosing regimens: (1): 2 g loading dose over 30 min followed by continuous infusion of 6 g/24 h; (2) 4 g loading dose over 30 min followed by continuous infusion of 6 g/24 h; (3) 2 g loading dose over 30 min followed by continuous infusion of 8 g/24 h.

**Table 6:** Probability of target attainment (PTA) ^a^ (in percentages) in the ascitic fluid between 24 to 96 h for the various temocillin dosing regimens according to the CL_CRurinary_ values for a target MIC of 8 and 16 mg/L.

Target MIC (mg/L)	CL_CRurinary_ (mL/min)	Temocillin Doses ^b^		
		Studied	Simulated	
		Regimen (1)	Regimen (2)	Regimen (3)
8 mg/L	20	98	98	99
	39.9	97	97	99
	60	96	98	98
	90	96	96	98
	120	87	87	94
	150	73	73	90
16 mg/L	20	93	95	96
	39.9	88	91	94
	60	76	80	90
	90	51	54	75
	120	32	33	56
	150	22	22	40

^a^ Dosing regimens achieving a priori target of PTA ≥ 90% are appearing on a green background; those achieving a PTA < 90% are on a red background. ^b^ Dosing regimens: (1): 2 g loading dose over 30 min followed by continuous infusion of 6 g/24 h; (2) 4 g loading dose over 30 min followed by continuous infusion of 6 g/24 h; (3) 2 g loading dose over 30 min followed by continuous infusion of 8 g/24 h.

## Data Availability

All data will be made available from the corresponding author upon request.

## Supplementary Materials

The following supporting information can be downloaded at: https://www.mdpi.com/article/10.3390/antibiotics11070898/s1: Figure S1: correlation between the concentration of total proteins and albumin in plasma and ascitic fluid; Figure S2: individual PK profiles of total and unbound temocillin in plasma and ascitic fluid; Figure S3: correlation between the penetration of temocillin in ascitic fluid an various parameters; Figure S4: correlation between the AUC of temocillin in plasma and ascitic fluid; Figure S5: Univariate association between the tested covariable and individual Bayesian estimates of PK parameter; Figure S6: Visual inspection of residuals plots in the plasma compartment; Figure S7: Visual inspection of residuals plots in the ascitic fluid compartment; Figure S8: PTA for temocillin in plasma and ascitic fluid at different CLCR_Urinary_ values and MICs; Figure S9: Graphical representation of the administration of temocillin for collection of samples; Table S1: Script of Pmetrics^®^ file for the final covariate model.
