# Supplementary material for: Population Pharmacokinetics of Temocillin Administered by Continuous Infusion in Patients with Septic Shock Associated with Intra-Abdominal Infection and Ascitic Fluid Effusion
Source: Antibiotics (Basel). 2022 Jul 5;11(7):898. doi: 10.3390/antibiotics11070898 (PMC9311849; doi:10.3390/antibiotics11070898)
Supplement: Supplementary file 1 [file antibiotics-11-00898-s001.zip › antibiotics-1766396-supplementary.pdf]

antibiotics

MDPI

Article

Population pharmacokinetics of temocillin administered by continuous infusion in patients with septic shock associated with intra-abdominal infection and ascitic fluid effusion

Perrin Ngougni Pokem, Xavier Wittebole, Christine Collienne, Hector Rodriguez-Villalobos, Paul M. Tulkens, Laure Elens, Françoise Van Bambeke, Pierre-François Laterre

Supplementary material

Table S1: script of Pmetrics® file for the final covariate model

```
#Pri (Primary variables)
V, 0.01, 50
CLi, 0, 6
K12, 0, 10
K21, 0, 10
K13, 0, 2
K31, 0, 2
V3, 0.01, 60
CL30, 0, 6
```

```
#Sec (Secondary variables)
Ke = CLi*(CLCRurinary/39.9)/V
K30 = CL30/V3
```

```
#Dif (Differential equations)
XP(1) = RATEIV(1) - (K12+K13+Ke)*X(1) + K21*X(2) + K31*X(3)
XP(2) = K12*X(1) - K21*X(2)
XP(3) = K13*X(1) - K31*X(3) - K30*X(3)
```

```
#Cov (Covariates)
CLCRurinary
```

```
#Out (Output equations)
Y(1) = X(1)/V
Y(2) = X(3)/V3
```

```
#Err (Error)
L=2.26
0.1,0.1,0,0
0.1,0.1,0,0
```

V (L), volume of the central compartment; CLi (L/h), population parameter estimate of temocillin clearance from central compartment; CL (L/h) typical estimate of clearance from central compartment;; K12 (h^{-1}), first-order rate constant for distribution from central to peripheral compartment 2; K21 (h^{-1}), first-order rate constant for distribution from peripheral compartment 2 to central compartment; K13 (h^{-1}), first-order rate constant for distribution from central to peripheral compartment 3 (ascitic fluid compartment); K31 (h^{-1}), first-order rate constant for distribution from peripheral compartment 3 to central compartment; V3 (L), volume of the compartment 3; CL30 (L/h), clearance from compartment 3 defined as the product between of first-order elimination rate constant from compartment 3 (K30 (h^{-1})) and volume of the compartment 3 (V3 (L)); XP(n), notation for dX(n)/dt where n is the compartment number; RATEIV(1), notation to indicate an infusion of drug (1); X(n), amount of drug in compartment where n is the compartment number; Y(1), concentration of unbound temocillin in the central compartment; Y(2), concentration of unbound temocillin in the compartment 3; Error, each observation is weighted by $1/(\text{Error})^2$ using a additional error model $\text{Error} = (\text{SD} + L^2)^{0.5}$, where SD is the standard deviation of each observation which is modelled by a polynomial equation (SD=C0+C1Y+C2Y2+C3Y3) with coefficients of the assay error specified in the bottom rows for unbound temocillin concentrations in plasma and ascitic fluid respectively, and L (lambda factor) is a value relating to extra process noise related to the observation, such as mis-specified dosing and observation times.

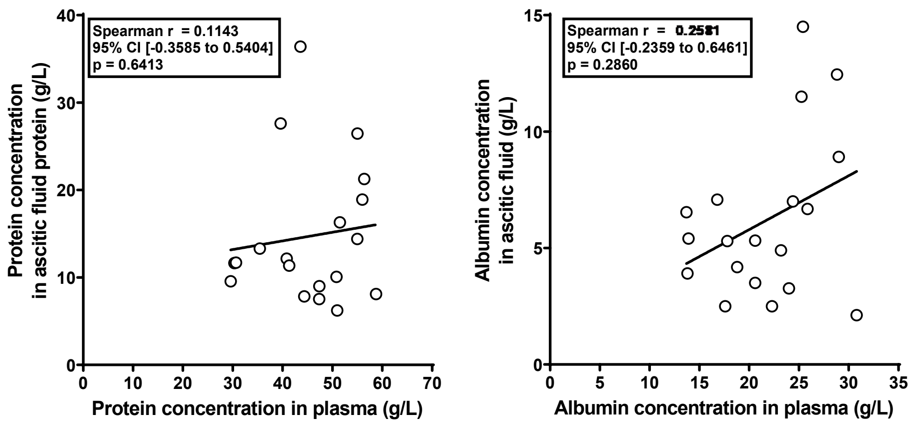

(a) (b)

Figure S1: correlation between the concentration of total proteins (a) and of albumin (b) in plasma and ascitic fluid as measured for the 19 patients included in the study. Spearman correlation coefficient with 95% confidence interval and values are shown, illustrating that the correlations were not significant.

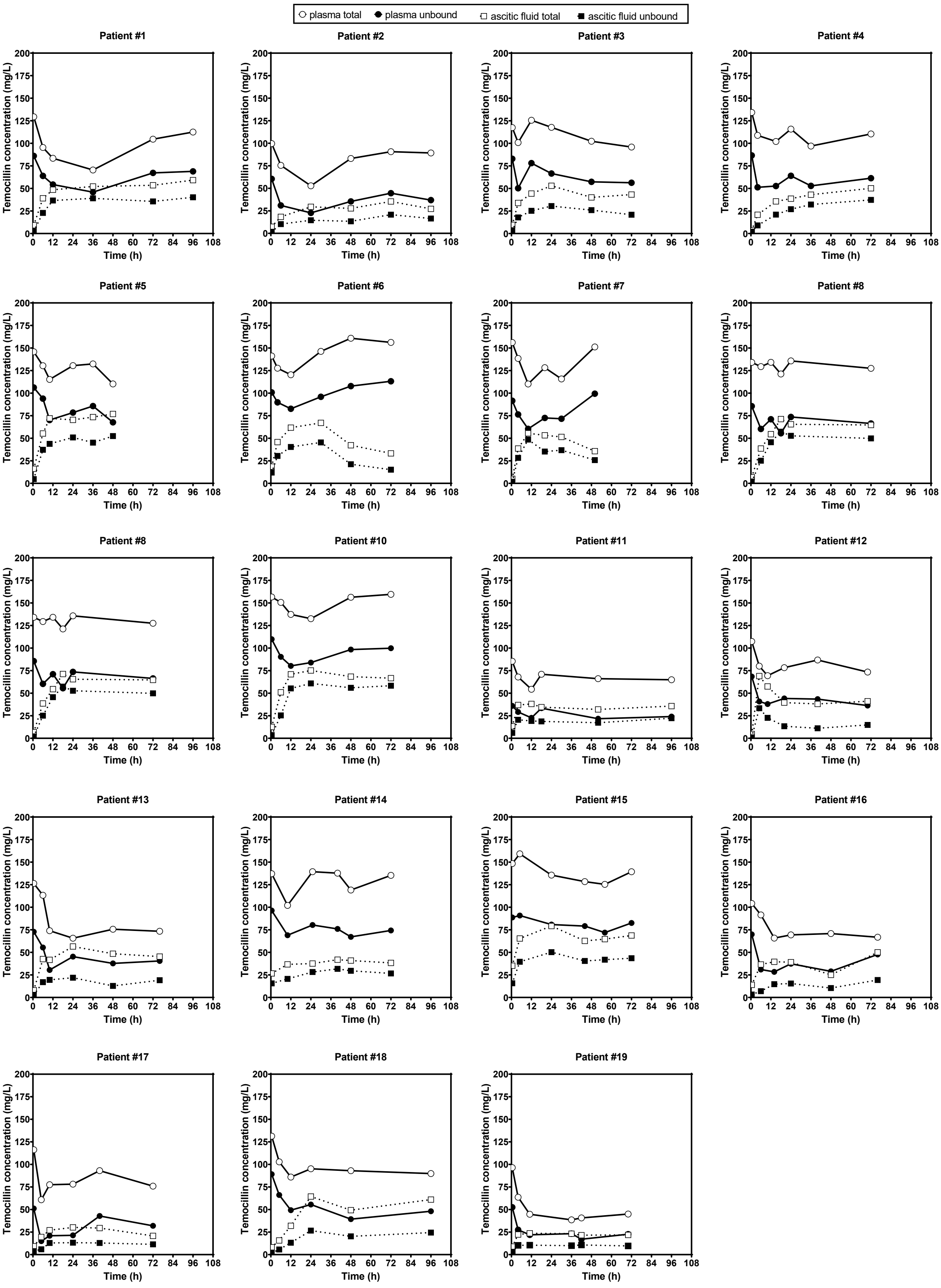

Figure S2. Individual PK profiles of total and unbound temocillin concentrations in plasma and ascitic fluid of septic patients with intra-abdominal infection and ascitic fluid effusion. All patients (N=19) received a loading dose of 2 g over 30 min followed by a continuous infusion of 6 g / 24 h.

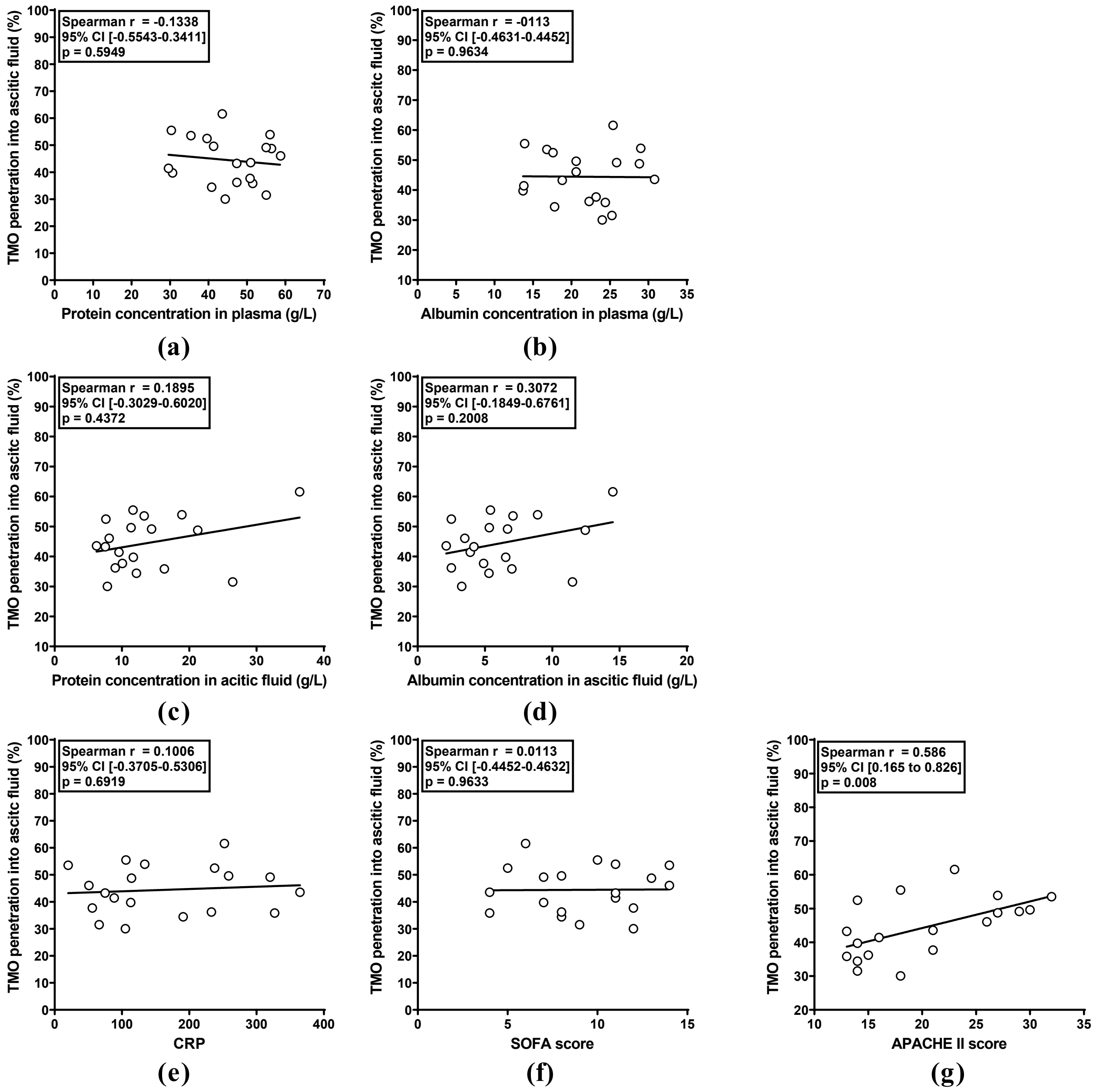

Figure S3. correlation between the penetration of temocillin in ascitic fluid and total proteins in plasma (a), albumin in plasma (b), total protein in ascitic fluid (c), albumin in ascitic fluid (d), CRP (e), SOFA score (f), and Apache II score (g) for the 19 patients included in the study. Spearman correlation coefficient with 95% confidence interval and values are shown, illustrating that the correlations was significant only for Apache II score.

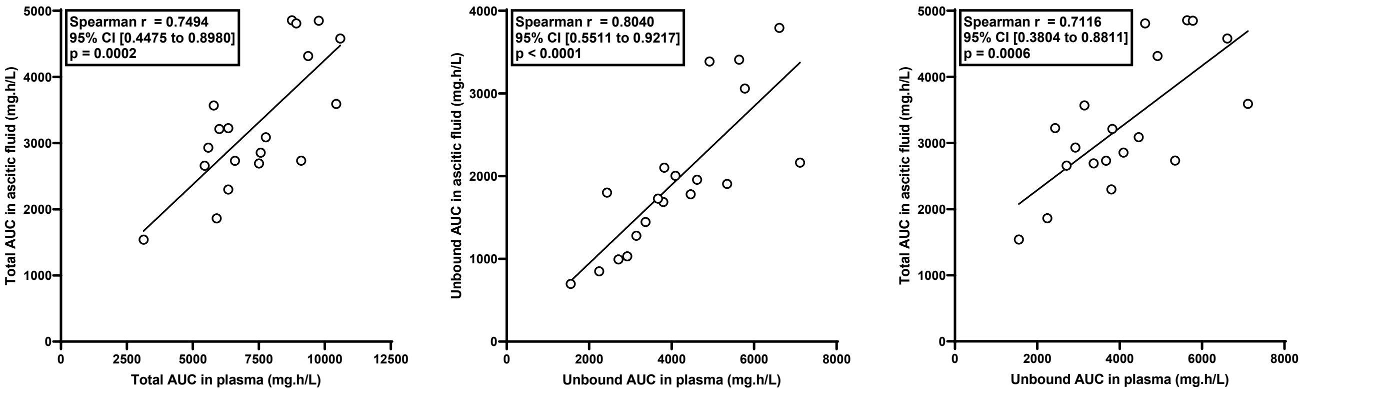

(a) (b) (c)

Figure S4. correlation between the AUC of temocillin in ascitic fluid and in plasma for total concentrations (a) or unbound concentrations (b) or between the AUC for unbound concentrations in plasma and total concentrations in ascitic fluid (c) for the 19 patients included in the study. Spearman correlation coefficient with 95% confidence interval and values are shown, illustrating that the correlations were highly significant.

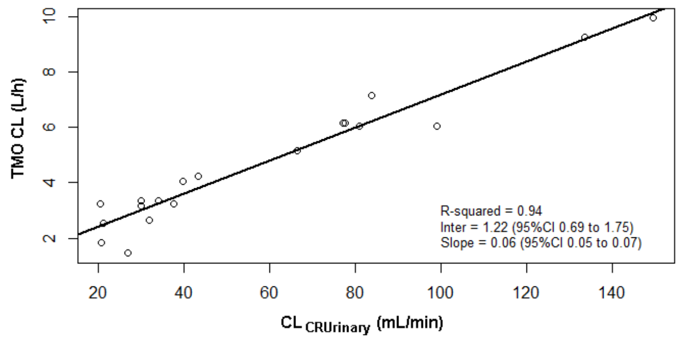

Figure S5. Univariate association between the tested covariate and individual median Bayesian estimates of PK parameter. Linear relationship between plasma clearance of unbound temocillin (L/h) *vs* measured urinary creatinine clearance ($CL_{CRurinary}$; mL/min). R^2=0.94; intercept = 1.22 [95%CI 0.69 to 1.75]; slope = 0.06 [95%CI 0.05 to 0.07].

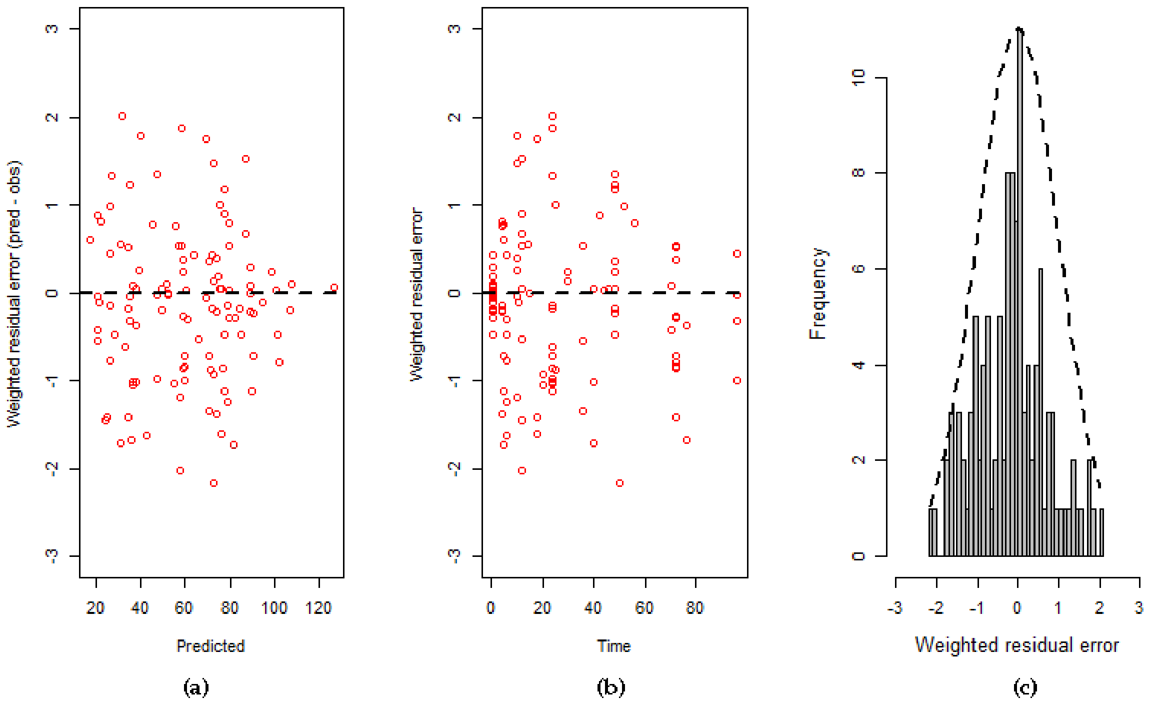

Figure S6. Visual inspection of the residuals plots in the plasma compartment. (**a**) weighted residuals error vs population predicted unbound temocillin concentration [Mean = 0.014 ±0.78; Student's t-test, p=0.096]; (**b**) weighted residuals error vs time [Mean = 0.14 ±0.78; Student's t-test, p=0.096]; (**c**) frequency distribution obtained from final model ([D'Agostino, p= 0.714]; [Shapiro–Wilk, p=0.514]; [Kolmogorov-Smirnov, p=0.796]).

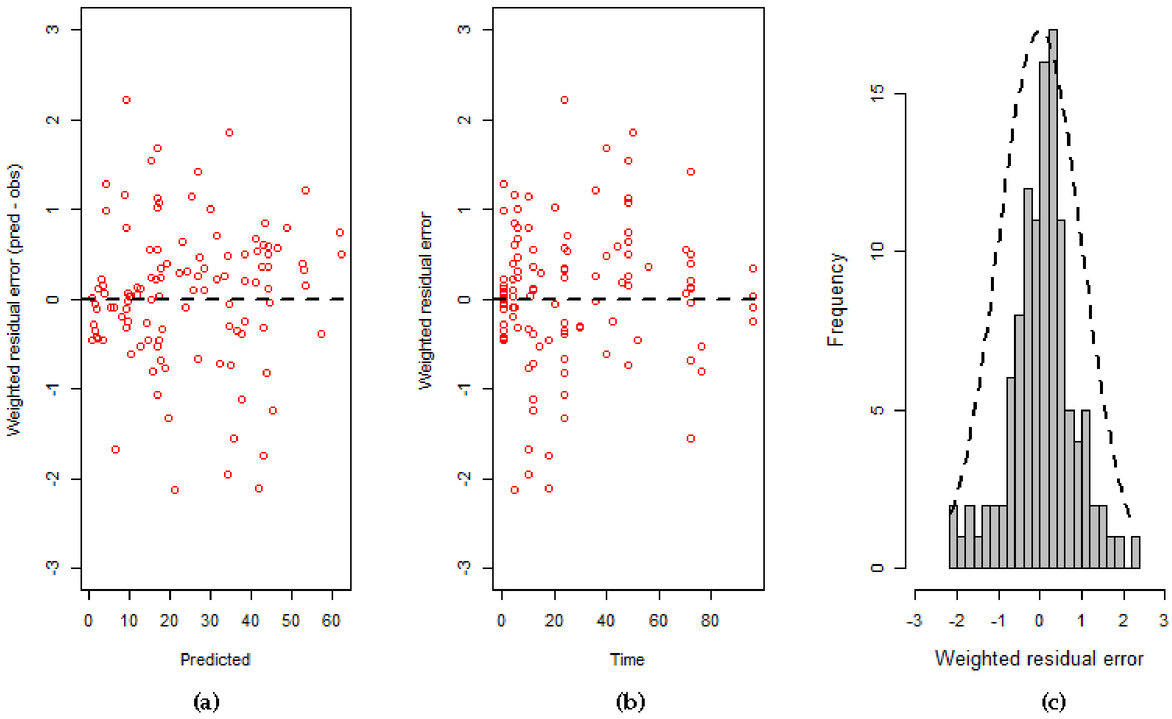

Figure S7. Visual inspection of the residuals plots in the ascitic fluid compartment: (**a**) weighted residuals error vs population predicted unbound temocillin concentration [Mean = 0.05 ±0.78; Student's t-test (p=0.478]; (**b**) weighted residuals error vs time; [Mean = 0.05 ±0.78; Student's t-test (p=0.478)]; (**c**) frequency distribution obtained from final model ([D'Agostino, p= 0.371]; [Shapiro–Wilk, p=0.041]; [Kolmogorov-Smirnov, p=0.797]).

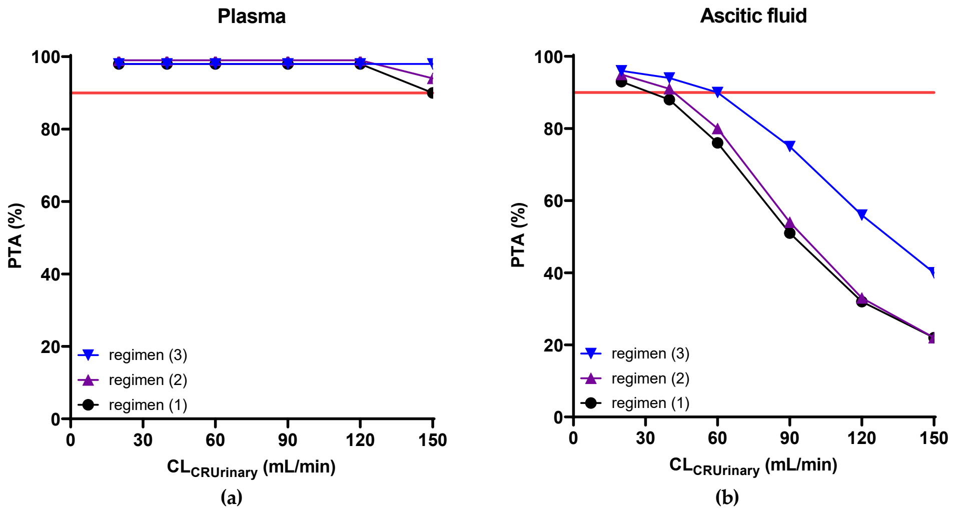

Figure S8. PTA for temocillin in plasma (**a**) and ascitic fluid (**b**) at different $CL_{CRurinary}$ values and MICs. Monte Carlo simulations dose regimens: (1) 2 g loading dose over 30 min followed by continuous infusion of 6 g/24 h; (2) 4 g loading dose over 30 min followed by continuous infusion of 6 g/24 h; (3) 2g loading dose over 30 min infusion followed by continuous infusion of 8 g/24 h. The PK/PD target is 100% *f*T> MIC; the PK/PD breakpoint corresponds to a PTA ≥ 90%; the target MIC is 16 mg/L.

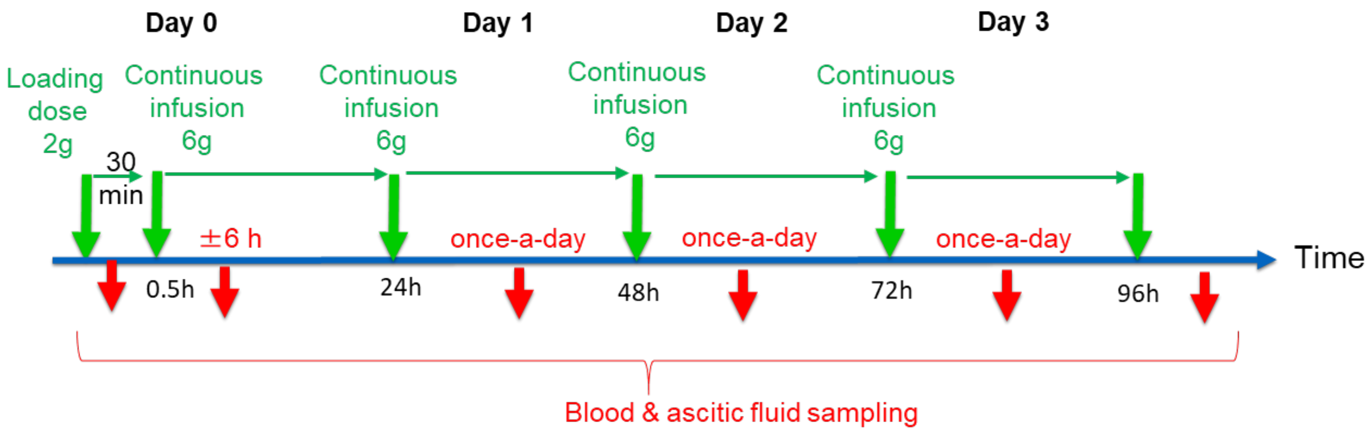

Figure S9: Graphical representation of the administration of temocillin to the patients for collection of blood and ascitic fluid to measure the total and unbound drug concentrations.
